# A study to enhance medical students’ professional decision-making, using teaching interventions on common medications

**DOI:** 10.3402/meo.v20.27097

**Published:** 2015-06-05

**Authors:** Jane Wilcock, Janet Strivens

**Affiliations:** 1 Faculty of Health and Life Sciences, Institute of Learning and Teaching, School of Medicine, University of Liverpool, Liverpool, UK; 2 Educational Developer, Centre for Lifelong Learning, University of Liverpool, Liverpool, UK

**Keywords:** critical thinking, professionalism, prescribing, antenatal pertussis vaccination, patient centredness, medical education

## Abstract

**Aim:**

To create sustained improvements in medical students’ critical thinking skills through short teaching interventions in pharmacology.

**Method:**

The ability to make professional decisions was assessed by providing year-4 medical students at a UK medical school with a novel medical scenario (antenatal pertussis vaccination). Forty-seven students in the 2012 cohort acted as a pretest group, answering a questionnaire on this novel scenario. To improve professional decision-making skills, 48 students from the 2013 cohort were introduced to three commonly used medications, through tutor-led 40-min teaching interventions, among six small groups using a structured presentation of evidence-based medicine and ethical considerations. Student members then volunteered to peer-teach on a further three medications. After a gap of 8 weeks, this cohort (post-test group) was assessed for professional decision-making skills using the pretest questionnaire, and differences in the 2-year groups analysed.

**Results:**

Students enjoyed presenting on medications to their peers but had difficulty interpreting studies and discussing ethical dimensions; this was improved by contextualising information via patient scenarios. After 8 weeks, most students did not show enhanced clinical curiosity, a desire to understand evidence, or ethical questioning when presented with a novel medical scenario compared to the previous year group who had not had the intervention. Students expressed a high degree of trust in guidelines and expert tutors and felt that responsibility for their own actions lay with these bodies.

**Conclusion:**

Short teaching interventions in pharmacology did not lead to sustained improvements in their critical thinking skills in enhancing professional practice. It appears that students require earlier and more frequent exposure to these skills in their medical training.

Professionalism defines the values of a doctor and is set out by the General Medical Council (GMC) for today's UK doctors (1); among these is ‘make the care of your patient your first concern’. Duties of a Doctor in Good Medical Practice (1) also states ‘you are personally accountable for your professional practice and must always be prepared to justify your decisions and actions’. This is further discussed, under Good clinical care 16e, in the ‘GMC document Medical students: professional values and fitness to practice’, which states ‘treatment should be based on clinical need and the effectiveness of treatment options, and that decisions should be arrived at through assessment and discussion with the patient’ (2).

Justifying decisions requires an understanding of contemporary knowledge, and whilst advances in medical understanding, therapies, and the world wide web have made large numbers of research articles available from around the world, it also makes individually knowing all available research, or even knowing best resources, less likely. For example, hypertension has been treated since the mid-1980s (3). A search on the university online library for terms ‘primary prevention of hypertension’ gave 13,426 results in 2014 and on ‘hypertension’ reveals 1,336,878 results. Most medical students still prefer to read textbooks in the UK, and there is understandable poor discrimination about which online resources they use (4). The development of internet-supported authorities, reviews, guidelines, and risk scores help to provide solutions to clinical scenarios, but at the expense of individual critical thinking skills, that is, students may develop a knee jerk learnt solution to a prescribing issue rather than learning to think through the problem. Maudsley and Strivens (5) noted that ‘British undergraduate curricula have long struggled to prevent factual overload from suppressing critical thinking’. Facione et al. (6) identified in professional decision-making ‘problem resolution’, which may be taught through guidelines, and ‘problem framing’. The student who has framed the wrong professional question may reach the wrong resolution. Rather than train students to every eventuality, university education should encourage students to ask professional questions of the specific context, so that students frame questions which become transferable across different scenarios. Facione identified critical thinking and inquisitiveness as two of seven personality traits associated with good skills in problem framing and problem solving, and these should be encouraged. Students need to think objectively, analyse evidence-based medicine (EBM), justify the beliefs they are developing, and share those with their peers. I share Winters and Echeverri's (7) view of the teaching of EBM which is described as: 1) ask the clinical question; 2) search for best evidence; 3) critically appraise the evidence; 4) integrate the evidence into practice; and 5) evaluate the outcomes.

Winters and Echeverri (7) identified barriers to teaching EBM as: lack of knowledge, belief, and skills regarding EBM; lack of critical appraisal skills; and taking too much on. Prescribing scenarios are particularly complex as there are medical system issues, for example, renal function, interactions, safety, convenience, monitoring, length of therapy, changes in patient conditions, balances between short- and long-term outcomes, costs, and patient views. After analysing information, students and doctors need to create outcomes. Outcomes are described by Porter (8) as inherently condition-specific, multidimensional, and factors requiring weighing against each other. This moves Winters’ EBM skills into ethical areas. For this study, I advocated the Beauchamp and Childress’ ethical principles (9) of beneficence, non-maleficence, autonomy, and justice because the students had been taught these in previous years.

Prescribing was chosen as the professional thinking exemplar because of increasing patient co-morbidities and polypharmacy, risking harms, side-effects, and interactions. Hagstrom et al. (10) surveyed patients and found that individuals declaring a chronic disease ‘increased from 23% in 1980 to nearly 40% in 2000’. The National Patient Safety Agency (11) reports that 6.5% of hospital admissions were related to medication issues, of which 9% were definitely preventable. Drugs most commonly associated with harms were aspirin, diuretics, warfarin, and non-steroidal anti-inflammatory drugs, usually causing gastrointestinal haemorrhage. The Medicines and Healthcare Products Regulatory Authority received 297 reports of fatality to warfarin (an anticoagulant) between 1963 and 2008 (12). About 700 deaths per year in the UK are considered to be directly attributable to medications and also preventable. The GMC publication ‘Good practice in prescribing and managing medicines and devices’ (13) aims to improve prescribing skills. In addition, a Prescribing Safety Assessment examination has been introduced to medical schools throughout the UK, so medical students should be gaining understanding of evidence for drug use, indications, and starting to critically assess their prescribing decisions.

It is on this background that I chose to discuss medications as a vehicle for improving professional decision-making. In creating future professionals, medical students are encouraged to act as active self-directed learners to build on prior learning, explore known concepts, and analyse new knowledge and experiences (14). The role of the tutor is to discover what the student already knows and then act as a mentor to provide ‘scaffolding’, which allows the students to work out best ways to learn autonomously. Using these principles in this study, the students were offered constructivist learning opportunities to progress from novice to life-long learners and improvers as clinicians.

## Methods

### Pretest and post-test study: student critical thinking using antenatal pertussis vaccination scenario

To explore approaches to problem solving with year-4 students in the 2012–2013 academic year, I gave them a short questionnaire exploring student attitudes and knowledge of antenatal whooping cough (pertussis) vaccination (Appendix 1). The questionnaire was wide ranging so that students would not try to predict tutor-wanted responses. The questions relating to the study are numbers 1, 2, 5, 6, and 12. Answering it was voluntary, 47 students returned the form and 1 student did not.

In April 2012 in the UK, the Health Protection Agency declared a level 3 incident response to rising levels of pertussis in neonates and recommended vaccination of women between 28 and 38 weeks of pregnancy with a vaccine containing pertussis, diphtheria, and tetanus from October 2012 (15). Antenatal vaccination has little precedent; in 2008, flu pandemic antenatal influenza vaccine was used, and postvaccination surveillance suggested a small risk of narcolepsy related to Pandremix vaccination in offspring. Little is known about actual risk, *in vivo* vaccination transfer from mother to foetus, and potential problems with adjuvants (16). Pertussis vaccine, given as Repevax, contains acellular pertussis, diphtheria, tetanus and polio, formaldehyde, glutaraldehyde, neomycin, streptomycin, polymyxin B, or bovine serum albumin. Antenatal vaccination with pertussis was therefore used as a scenario to which students would not have an ‘off the peg’ response, guideline, app., or e-resource readily available. This pretest study was in conjunction with another clinical tutor to gain necessary numbers. At the next meeting, students were asked; ‘How do you feel about performing a new skill?’ and ‘What would you want before doing a new skill?’ in order to encourage a discussion of attitudes and critical thinking – themes were then brought together by the two tutors.

This same questionnaire and discussion was undertaken as a post-test study in the 2013–2014 medical student group but only by one tutor, 8 weeks after the prescribing interventions and the 2-year group results compared. The expected outcome was that the second group would display improved critical thinking and professional questioning.

### Prescribing interventions

The intervention group was six groups of 6–10 fourth year medical students whom I tutored for 1 day every 2 weeks throughout their academic year 2013–2014. At three meetings, 40-min teaching topics were introduced on the commonly used medications: aspirin, tiotropium, and simvastatin (Appendices 2–4). The teaching was subdivided into four parts:

1. Tutor and student discussion of facts about the chosen drug. A table of important facts was established to improve and uniform baseline knowledge.
2. Tutor questioning about drug effectiveness in a specific common scenario to verbalise drug efficacy beliefs among students. Presentation of a major trial in abbreviated form, outlining method of study, with headline outcome numbers of benefits and harms. Students were asked to respond to the EBM information.
3. Students were asked ‘what other considerations are there in prescribing, beyond EBM, and the facts about the drug?’ This was frequently rephrased, due to lack of response, to ‘can you tell me any ethical principles that could be used in deciding whether to prescribe?’ The students recalled the Beauchamp–Childress four ethical principles and were encouraged to relate these to the medication.
4. A variety of scenarios were discussed in which different prescribing judgements might be made with the understanding that no one answer would always be right but that there was an effort at best professional judgement.

The three scenarios used were: aspirin after a heart attack, the use of tiotropium handihaler in chronic obstructive airways disease (COPD), and use of simvastatin to reduce cholesterol in patients with coronary heart disease (CHD). Over the 6 weeks, students became accustomed to the structure of the teaching, to critically analysing EBM, and discussing ethical aspects of prescribing.

To offer a variety of tutor methods to allow students to find EBM resources, practice critical analyses, and to model their behaviour on my interventions, I asked a student in each group to volunteer to tutor on another medication of their choice, or suggested by myself. I helped students find respected EBM resources online if asked. They were asked to follow the same tutoring format. Each of the six groups, therefore, had a further three teaching interventions over 6 weeks but by peers. A total of six medications were discussed over the study for each group.

During student presentations, I made notes using a table to note if EBM and ethical issues had been discussed. I was keen that students remained autonomous thinkers and that I did not create another ‘guideline’ on how to think so I encouraged questions and problem framing rather than answers.

After the six interventions there was an 8-week gap in which we did not discuss EBM and critical thinking unless initiated by students. I then administered the post-test part of the study as above.

The study had ethical approval from the university (study 201208117). Students were not consented into the study for two reasons: first, it was impossible if students did not consent to exclude them from participation as the study took place in usual teaching sessions. Second, if students were consented into a study to look at ethical behaviour they might perform to the study outcomes rather than offer true opinions of patient problems and management. At all times, participation in discussion, teaching peers, and filling in the final questionnaire was voluntary among the students.

## Results

### Prescribing interventions

When students asked about drug information, they wanted information as found in the British National Formulary (BNF). When asked about further information they asked about serious interactions, illnesses that they should aware of when prescribing, overdose, effects in pregnancy, and cost. Students did not ask about EBM of action, EBM of benefits, or harms initially; although they learnt this over the three cycles and were interested.

Students were asked how effective they thought the drug was in the scenario and were encouraged to guess. The figure for benefit of aspirin in preventing further heart attack or angina in secondary prevention patients was estimated as highly effective, mode 70% effective – when encouraged to round down the lowest guess was 17%. Students were very surprised at the lack of magnitude of efficacy of aspirin and other medications. They vastly overestimated effect and underestimated harm. Reactions to this were, ‘so medications aren't as good as we thought they would be’ and ‘I wouldn't take it’.

Students enjoyed talking about trials but were not used to handling information and made simple errors in summating patient numbers, dropouts, and percentages and were unable to critique studies accurately. After the first session, I gave them printouts so that they had the figures before them as well as on the white board and could take them home.

Students did not consider ethical principles, for example, benefits, harms to patients and society, and autonomy without prompting. They struggled to use these in an abstract manner and improved when patient contexts were used. When initially asked to consider ethical principles in prescribing, two comments were, ‘Oh, is this an ethical question then?’ and ‘Is it the society, groups, and individuals stuff?’

Students chose to present on amoxicillin, ramipril, metformin, citalopram, ibuprofen, and amlodipine. Students generally followed the tutor format but it was unusual to have the evidence well-presented, and errors were common. Students suggested resources like National Institute for Clinical Excellence (NICE) clinical guidance, Health and Social Care Information Centre, found their own references, and went to a lot of trouble to review the information. Interpretation was variable, and some student-tutors could not distinguish absolute risk reduction from relative risk reduction, so overestimated drug effects.

Students used PowerPoint and handouts. Some students rejected suggested trials in favour of other meta-analyses, one student introduced Forest Plots. Two students introduced groups to Patient Decision Aids. A few students used numbers needed to harm (NNH) and numbers needed to treat (NNT) as markers of effect. One student discussed with the group about clinical trials and lack of grey (unpublished evidence) and called on the group to sign the petition at www.alltrials.com. Students found Patient Decision Aids, NNH, and NNT most effective.

Student-tutors who presented varying patient scenarios created more discussion with peers about harms and benefits than those who did not contextualise prescribing scenarios.

Students had difficulty formulating ethical questions. Student-tutors and I resorted to asking the group personal questions, for example, ‘What if I were to give you this drug tomorrow?’ and ‘What if the patient says, do I really need to take this drug, doctor?’ Only one student discussed that a 3% reduction in death from heart attack was highly significant across the UK population, others were likely to say, ‘Well, I wouldn't take it’. One student suggested placebo drug use. Most students wanted to prescribe amoxicillin despite contrary evidence of benefit. Responses to not prescribing amoxicillin were ‘get a good lawyer’, ‘explain the EBM’, ‘get a sputum sample or a CXR’, ‘stop doing medicine’, ‘prepare for aggression’, ‘go to Spain and stock up’, and ‘self-treat with home remedies’. There was some discussion about public health campaigns. In discussing autonomy and amoxicillin use, one student told the group, ‘I would tell them they had to take it or they would die’. When asked if that would be true the reply was, ‘Not really’. A response in relation to ethics was, ‘It's really confusing’. There was therefore concern that unless guidance was followed patients might litigate or complain. Some students’ prescribing preferences were directed by their own illness experience despite the evidence. A discussion about not prescribing creating patient aggression led to a student-observed anecdote of a patient becoming aggressive in a GP consultation. Students lacked non-prescribing consultation skills and management plans and wanted to share concerns about complaints.

During the gap of 8 weeks, I did not discuss prescribing issues, EBM, or ethics unless they came up in case presentations. On one occasion, a student gave a patient-centred presentation but did not connect the patient's warfarin (an anticoagulant) prescription to the patient's complaint of haemoptysis: this student had not critically assessed the patient's medications. Students did not continue to discuss EBM or ethics in presenting case histories. Compared to previous years, however, I found the case presentations contained more patient information about function and views.

### Pretest and poststudy questionnaires

In the pretest study, 47 students handed back the questionnaire, one did not.

In the post-intervention group after 8 weeks, 42 students (six students were absent due to illnesses) were asked to complete the questionnaire and they all handed them back. The full results of the 2012–2013 and 2013–2014 year groups’ responses are in Appendix 1. The results relevant to the prescribing intervention are in Table 1.

**Table 1:** Results of survey into student attitudes to antenatal pertussis vaccination pretest and post-intervention groups

Question number and response	Statements from questions	Non- intervention group (pretest);% of students choosing these options (total number of students: 47)	Post-intervention group (post-test);% of students choosing these options (total number of students: 42)
1. parts 3 and 4	Dissatisfied with their knowledge of vaccination of pertussis in pregnancy	91	95
2. parts 1 and 2	Would vaccinate in pregnancy	19	24
2. part 3	Would not be confident to vaccinate but would do so after checking with nurse or online as it is a government recommendation	68	64
5. parts 1 and 2	Had concerns regarding maternal harm in vaccination	4	31
5. part 3	Were unsure whether they had concerns or not, about maternal harm	43	55
6. parts 1 and 2	Had concerns about harms to the baby	30	29
6. part 3	Were unsure whether they had concerns or not, about harm to the baby	47	55

Responses to question 1 confirmed that this was a novel scenario for these medical students, which was the intention of the study. Ninety-five percent of students post interventions were dissatisfied with their knowledge of antenatal pertussis vaccination yet 88% (question 2 parts 1, 2, and 3) would give the vaccination; this figure is 87% in the pretest group. The only clear disparity between the pretest and post-intervention students’ responses was that post-intervention students were more likely to have concerns about maternal harm. There was some small change in post-intervention students being less certain about concerns of foetal harm in vaccination. In the free text boxes, students from both groups commented that they knew nothing about pertussis vaccination in pregnancy and would like to know more. The preliminary group made 17 comments and the study group made 28 comments (question boxes 1, 2, 5, 6, and 12, Appendix 1), a number commenting that they required more information in order to make decisions. Nevertheless in both groups, despite misgivings, students overwhelmingly would give the vaccination if asked.

Two weeks later the post-intervention students were given the results above and asked two questions ‘How do you feel about performing a new skill?’ and ‘What would you want before doing a new skill?’

All groups were clear that they would trust the expert asking them to give the vaccine. Responsibility for long-term side-effects due to vaccination would lie with guideline authorities. A few students mentioned risks of anaphylaxis but, when asked if they knew if adrenaline for anaphylaxis would be present, said they would not check but assumed so. They stated that anaphylaxis was rare and that the nurse would be trained to manage it. Rates of anaphylaxis are rare at about 1 in 1,000,000 vaccinations (17). Students were pleased to take on new skills, though occasionally anxious, but did not feel a need for EBM or ethical consideration. Comments made were ‘we are asked to do new things all the time so just get on with it’. This response was exactly the response of the pretest student groups. The only difference was that some of the post-test students stated that ‘I know it's wrong and we should think about the vaccination but in practice we don't’.

Therefore, short teaching interventions in pharmacology did not lead to clear sustained improvements in medical students’ critical thinking skills in enhancing professional practice. Barriers to conversations about professional decisions related to poor EBM interpretation skills, reluctance to change practice, and not recognising common prescribing scenarios as having ethical dimensions.

## Discussion

This study confirmed that professional thinking skills need to be taught explicitly in the medical curriculum at an early stage and be developed over the years. Donaldson et al. (18) reported on students who had been asked to bring ethical cases for discussion and found that many focused on legal obligations rather than the morality of what should be done. In addition, he found students identified ethical issues as occurring at times of conflict in medical management and that students can describe ethical issues but were not used to applying them in decision-making. This is in agreement with this study. Students require practice in logic and reasoning skills and placing them in a value-laden practice, which is patient centred; this could be encouraged by experts vocalising their decision-making processes more explicitly. The apprentice – master model of clinical placements in undergraduate medical education – relies on students mimicking expert behaviour. Students are rarely asked their views in scenarios (particularly when best practice is not obvious or not guideline driven), have intellectual conversations about best practice, or allowed to propose alternative management plans.

A number of student-tutors emailed in advance or asked in the presentation if they were ‘doing the right thing’. Freeing students to appreciate that there was no right and no wrong answer to problems led to an increase in maturity of discussion with consideration of patient values in case and topic teaching discussions until the end of the year.

The study was limited by time, the intervention was limited to 40 min and this particularly limited the feedback that could be given to the student-tutor contributors, and general end-of-session feedback to emphasise learning points or provoke further reflection. There was only myself delivering the study and assessing the outcomes; this study would have been much stronger with peer-tutor review and a peer-tutor in class observation making a note of emerging themes. There may have been effects of my own values on topic teaching and interpretation of comments, which might affect the validity of results and identification of themes. It was difficult to listen to students facilitating the group, respond when required, and make observational notes about content. Using direct quotations allowed some accurate recording of activity. A form created to record student activity was not easy to use or adequate in recording themes.

The study benefitted from using small groups who had already met on five occasions with tutor-led and peer-peer teaching of topics, so the study slotted into the day. As there was already trust between tutor and small groups, the reflections were honest. Starting the study towards the end of the first term meant that stretching the student learning to encompass professional thinking felt natural over the year. Having all students blinded to the study meant that discussions were frank and honest and is a major strength of this study. In developing a number of teaching interventions, the students were able to evaluate medications, scenarios, online resources, and practice skills improvement over a number of months.

The choice of antenatal pertussis vaccination could be criticised as relating professional thinking to a skill in which instructional method might be more usual but I believe that all medical actions should belong to a reflective practitioner who is able to consider whether undertaking the procedure is in the best interests of the patient. In setting up professional scenarios, practice becomes quickly outdated and tutors require signposting to resources to get started and need to create a repository of respected resources, clinical cases using context, information, and ethical aspects, to create decisions and quality assurance and reflection of these.

## Conclusion

In conclusion, students were not able to transfer skills developed in professional decision-making to a novel medical scenario. They have no in-depth knowledge of likely incidence or prevalence of harms and benefits of commonly used medications and so made highly inaccurate assumptions. Medical students need to contextualise facts, evidence, and ethical information and consider patient individual factors before arriving at decisions. It may be that students are able to consider ethical issues but reserve judgement without more information and find abstract decision-making unsatisfactory. The early development of professional questioning would help students to mature. I think that tutors should encourage experimental thought and error to gain best ways of decision-making and best decisions which then translate into good decisions in clinical practice, for example, good prescribing decisions. Professional questioning would allow students to have an overview of medical management. In this study, medical students were unable to progress in answering therapeutic questions as they hesitated at the EBM content, lacked skills in interpretation and were then reluctant to discuss an overview of patient prescribing without this. This inability to develop overarching professional views meant that most students were willing to give vaccinations in novel circumstances (pregnancy) despite a lack of knowledge and were not willing to take responsibility for their decision.

Skills training and resource pointing to interpret EBM to decide best evidence in the specific patient circumstances could be fixed within a framework of individualised patient scenarios with ethical-based values in order to foster open appraisal of benefits and harms which might run counter to preconceptions. This requires time to let students practice skills over a number of scenarios during all of their training – in this way, students can choose to develop attitudinal and epistemological change. Students should be encouraged to explore their understanding that outcomes can be uncertain; influenced by evidence, context, and patient choice; and be multiple. This requires freeing up medical curriculums into less content-heavy syllabuses and allowing experimentation of ideas in class and on clinical placements. This requires a change in tutor culture and timing; in practice, it is easier to train a medical student to identify a problem and select a known accepted response. Is this what society would like from its doctors? Does it provide the best care? If it does then it is possible that medical school training could be provided as a distance learning knowledge base and later clinical expert–apprentice model.

## Appendix 1

Responses to Whooping cough vaccination in pregnancy questionnaire year 4 medical students

Pretest year 2012–2013 in bold and post-test study group 2013–2014 cohort in italics

Whooping cough vaccination in pregnancy. There were **47/48 replies** *42/48 replies*

Please assume your area GP StR1 and a pregnant woman attends your next surgery session when replying.

1. Do you feel knowledgeable about whooping cough vaccination in pregnancy? Please delete the responses not applicable leaving the response you most agree with.

**Table:**

	Pretest	Post-test
1. I am very knowledgeable about whooping cough vaccination in pregnancy		
2. I am satisfied with my knowledge about whooping cough vaccination in pregnancy	**4**	*2*
3. I am not satisfied about my knowledge about whooping cough vaccination in pregnancy	**23**	*19*
4. I feel I lack knowledge about whooping cough vaccination in pregnancy	**20**	*21*
**Pretest comment ‘I am unsure about the timings’**		
*Post-test comments ‘I do not know if it is safe in pregnancy’* *‘I do not know anything about whooping cough vaccination in pregnancy’* *‘I have never heard of it’* *‘I am unsure about time-frames and risks’*		

2. What is your personal position about vaccinating a pregnant woman against whooping cough?

Please delete the responses not applicable leaving the response you most agree with.

**Table:**

	Pretest	Post-test
1. I have already personally vaccinated a pregnant woman against whooping cough myself		*1*
2. I am happy to vaccinate the pregnant woman myself but have not yet had an opportunity to do so	**8**	*9*
3. I would not feel confident in vaccinating the pregnant woman myself but would do so after checking details with my nurse or online as it is a government recommendation	**32**	*27*
4. I am not happy about vaccinating a pregnant woman against whooping cough at present	**5**	*5*
5. I would not vaccinate a pregnant woman against whooping cough	**2**	
**Pretest comments: ‘Is it needed if the mum has had childhood vaccination?’** **‘I am unsure about the indications or reasons not to vaccinate’** **‘I am not confident as I am not sure it is safe?’** **‘It is not a live vaccine so I am happy to vaccinate’**		
*Post-test comments: ‘I would check with an expert and contact the national vaccination programme to gain advice’* *‘I would vaccinate if my GP or nurse tells me it is standard practice’*		

3. Should women post-partum be vaccinated against whooping cough?

Don't know=**23**. *11* Yes=**12**. *14* No=**4** *11*

Comments: **‘I would have to look this up’**

**‘I have no knowledge re evidence for efficacy’**

‘The benefit is for the baby so there is no benefit in giving post-partum vaccination to the mother’

‘Vaccination should be given if there is enough evidence that it is beneficial and cost effective, etc.’

4. a) Do you understand the mechanism of vaccination in pregnancy?

Don't know=**4**. *3* Yes=**11**. *11* No=**24**. *26*

b) Does the vaccination cause maternal antibody production and transfer of these antibodies to the foetus or does it cause the foetus to produce antibodies?

Maternal antibodies are transferred=**17**. *23* Don't know=**18**. *14* Both=**1** Foetal produces antibodies=**3**. *3*

Comment: **‘I would have to look this up’**

c) Would an understanding of the science of antibody transfer and production across the placenta be something you would be interested in?

Yes=**35**. *38* No=**5**. *3* Maybe=**2**

Comments: *‘I definitely need more teaching on this’*

‘I am a bit interested but only in the clinical relevance’
*‘This is an important topic and with anything in medicine patient lives are at risk therefore doctors should always consider the consequences’*.

5. Have you any concerns about harm to the woman? Please delete the responses not applicable leaving the response you most agree with.

**Table:**

	Pretest	Post-test
1. Yes I have a lot of concern about harm to the woman	**2**	*6*
2. Yes I have some concerns about harm to the woman		*7*
3. I am unsure whether there may be harm or not to the woman	**20**	*23*
4. I am fairly sure I have no concerns about harm to the woman		*5*
5. I have no concerns about harm to the woman	**5**	*1*
**Pretest comments: ‘There may be a risk of flu like illness and anaphylaxis’** **‘There may be a risk of flu and anaphylaxis like any vaccination’** **‘It might depend if it is a live vaccine (I have some concerns)’**		
*Post-test comments ‘I do not know what harm the vaccine can cause and whether this would be different in a pregnant woman’* *‘I do not know about vaccinations in pregnancy’*		

6. Have you any concerns about harm to the baby? Please delete the responses not applicable leaving the response you most agree with.

**Table:**

	Pretest	Post-test
1. Yes I have a lot of concern about harm to the baby	**4**	*7*
2. Yes I have some concerns about harm to the baby	**10**	*5*
3. I am unsure whether there may be harm or not to the baby	**22**	*23*
4, I am fairly sure I have no concerns about harm to the baby	**10**	*7*
5. I have no concerns about harm to the baby		
**Pretest comments: ‘I think there is a very small risk to the baby as with any vaccination it is not 100% safe’** **‘I would need to read more about it’**		
*Post-test comment: ‘I do not know if it is harmful to the baby or not’*		

7. a) Do you think GPs are best placed to vaccinate pregnant women?

Yes=**31** *32* No=**3** *3* Don't know=**4** *2*

b) Who do you think should be doing this procedure?

Don't know=**3**. *2* Midwife=**9**. *7* Practice nurse=**15**. *12* GP=**18**. *26* med student=**1**. Obstetrician=**2**. *5* Health Visitor=**1**. Paediatrician=*1* anyone appropriately trained=*3*

Comment: *‘I think midwives would be more cost effective than GPs’*

8. State what you think is the national uptake rate of whooping cough vaccination in pregnancy at present in%?

**5–90% range**. *5–80% range*. 5–10%=*5*. 15–30%=**14** *16* 31–50%= **5** *3* 51–60%= **5** *5*

9. If you were to vaccinate in your next surgery what is the name of the vaccine you would use?

Don't know=**30** *46* DTP and pertussis=**1**. Varied incorrect=*5*

10. Have you done any routine baby immunisations in the last 12 months? Y=**7** *3* No=**33** *36*

11. Would a pregnant woman attending you be given written information about whooping cough vaccination? Y=**25** *34* No=**2** Don't know=**13** *8*

12. Please write any comments below which would help me in understanding student thoughts, feelings and approaches to whooping cough vaccination in pregnancy including any further training you may have wanted.

**Pretest comments:**

 **‘I think more teaching should be given on issues such as this which change and have a big impact’**

 **‘I have received no formal teaching on this but know it is offered but not sure when’**

 **‘The only contact/info I have had re this has been through personal attendance at a GP surgery when I picked up a leaflet and read it’**

 **‘I don't have any knowledge of this’**

*Post-test comments:*

‘I know very little about this and would like to know more’
‘I don't think medical students can give vaccinations’
‘I don't know much about it’
‘This is an area I have little knowledge of’
‘I would like to have more information on the effects of vaccinations in pregnancy in general, not only whooping cough’
‘I have no knowledge so do not feel I can give the vaccine’
‘I have no idea what this is and want to know the benefit to the baby mainly’
‘I know very little about this’
‘I do not know enough about whooping cough vaccination in pregnancy to make decisions upon giving it or not giving it’
‘I need more education surrounding this’
‘If I knew more about it and there was the right evidence I would be happy to give it’
‘I have never heard of it’
‘I honestly don't know anything about this topic’

Thank you.

## Appendix 2. Tutor sheet regarding aspirin (November 2013)

*Should patients who have had a heart attack take long-term aspirin?*

At present students would be considered to be ‘doing well’ if they identified that the UK guideline is to provide patients with aspirin after a heart attack (MI) and be able to write this prescription safely, identify that it may cause wheeze or peptic ulceration and understand that it acts as an antiplatelet.
During a short teaching intervention students will be guided to consider the patient perspective:
Consider BNF facts about aspirin
How useful is it to the patient?
What are the risks of harm to the patient?
Drug use is considered in terms of Beauchamp and Childress’ 4 ethical principles:
In order to consider ethical issues students require more than guidelines, they require an evidence base. Evidence bases can found in NICE full guidelines and patient decision aids, also provided by NICE (previously by the National Prescribing Centre). There are other useful sources which students may subsequently research.
So for the scenario above the evidence would be:
Do good for the patient: Aspirin is known to reduce death from MI and cardiovascular events in patients who have had a MI. A 1970s study suggested that placebo (no aspirin or anticoagulant) patients had a death rate of about 8.5% over an average follow up of 22 months, reduced to 5.8% by quite high dose aspirin.
Do no harm to the patient: The CURE study had a rate of major bleeding in non-ST elevation (less severe) MI patients of 2.7% on aspirin. Students may want to think about definitions of major bleeding; general non-adherence rates for patients on long-term therapy and reasons for these; changes in patients’ susceptibility to harms over their lifetime.
Patients’ informed decision-making: Given the evidence, how would the students as doctors present the evidence to a patient? Is the evidence what they would have expected themselves?
Justice to the patient and to society: Students and doctors overestimate the benefits of medications at an individual level but a nearly 3% reduction in second MI and angina after a first MI throughout society (28% of all deaths in the England and Wales in 2012 were due to CHD and stroke) is very significant.
The students would be signposted to relevant resources by the tutor initially.

## Appendix 3. Tutor sheet regarding tiotropium (December 2013)

*How useful is tiotropium for patients with COPD?*

At present students would be considered to be ‘doing well’ if they identified that the UK guideline is to provided patients with tiotropium with COPD and be able to write this prescription safely, identify that it may cause a dry mouth and understand that it acts as an anticholinergic/antimuscarinic drug.
During a short teaching intervention students will be guided to consider the patient perspective:
BNF facts
How useful is it to the patient?
What are the risks of harm to the patient?
Drug use will be considered in terms of Beauchamp and Childress’ 4 ethical principles:
Facts: indication: maintenance COPD
Cautions: if eGFR < 50 ml/min, benign prostatic hypertrophy (BPH), bladder outflow obstruction, susceptible to acute angle eye glaucoma.
Side-effects: dry mouth, constipation, tachycardia, cough, paradoxical bronchospasm, palpitations, AF, headache, dizzy, urinary retention, blurred vision, dyspepsia, taste disturbance.
Interactions: discuss liver metabolised drugs
In order to consider the ethical issues here the students require more than guidelines. They require an evidence base. The evidence bases can found in NICE full guidelines and patient decision aids, also provided by NICE (previously by NPC). There are of course other sources that may be useful and it will be up to students subsequently to find best evidence.
So for the scenario above the evidence would be:
A long-term evaluation of once daily inhaled tiotropium in COPD; R. Casaburi et al. European Respiratory Journal Feb1, 2002, vol. 19 no 2 p. 217–224.
This is a major reference used in initial decision-making by NICE in including tiotropium in COPD guidance.
British Thoracic Society (BTS) guidelines also refer to NICE guidance.
This was a double-blind placebo randomised trial of inhaler without tiotropium and inhaler with tiotropium
921 patients ages 65 ±9 years with stable COPD. Is this reasonable?
Exclusions: last 4 weeks patients had had an exacerbation of COPD or a MI in the last 1 year of congestive cardiac failure (CCF) last 3 years or had a heart arrhythmia on medication. Is this reasonable?
Mean screening FEV1 was 39.1% vs. 38.1% of predicted
Measured changes were in FEV1, SOB, health status, medication use and adverse effects
81% tiotropium and 72% of placebo patients completed the study
9.6% tiotropium patients stopped due to adverse effects versus 13.7% of placebo.
2.4% tiotropium patients stopped due to lack of efficacy and 7% in placebo
There was less wheeze, superior health scores, fewer COPD exacerbations, fewer hospitalisations.
There was no difference in cough or chest tightness.
Side-effects: Tiotropium dry mouth 16% and 2.7% in placebo, other side-effects were at similar rates.
Action effects: morning baseline placebo PEFR: 190 increased to 205 at 1 year and evening PEFR 205 L increased to 210 L. Tiotropium baseline morning PEFR was also 190 L and improved at 1 year to mean 230 L and evening 215 to 240 L.
Bottom line: dry mouth 16% and 30 ml improvement in PEFR.
How does that make us feel about the medication? Is it worth giving? Is it worth pushing the patient if they don't want another inhaler?
Do good for the patient: improved PEFR and other scores. Mean improvement 30 ml so modest.
Do no harm to the patient: 16% dry mouth
Patients’ informed decision-making: Given the evidence, how would the students as doctors present the evidence to a patient? Is the evidence what they would have expected themselves?
Justice to the patient and to society: Students and doctors overestimate the benefits of medications at an individual level but a 30 ml gain in PEFR may be helpful to individuals: it also demonstrates how irreversible COPD is.
The students would be signposted to relevant resources by the tutor initially.

## Appendix 4. Tutor sheet regarding simvastatin (January 2014)

*Should patients who have had a MI or angina take simvastatin 40 mg one at night?*

At present students would be considered to be ‘doing well’ if they identified that the UK guideline is to provided patients with a statin after a MI and be able to write this prescription safely, identify that it may cause muscle pain, is liver metabolised and understand that it acts at the liver to reduce cholesterol.
During a short teaching intervention students will be guided to consider the patient perspective:
BNF facts
How useful is it to the patient?
What are the risks of harm to the patient?
Drug use will be considered in terms of Beauchamp and Childress’ 4 ethical principles:
In order to consider the ethical issues here the students require more than guidelines. They require an evidence base. The evidence bases can found in NICE full guidelines and patient decision aids, also provided by NICE (previously by NPC). There are of course other sources that may be useful and it will be up to students subsequently to find best evidence.
So for the scenario above the evidence would be:
Do good for the patient: Reduces second MIs and acts in primary prevention to reduce CHD.
Do no harm to the patient: acts as a competitive inhibitor of HMG CoA reductase, an enzyme involved in cholesterol synthesis especially in the liver so has a number of liver metabolised drug interactions
Indications: primary hypercholesterolaemia, prevention of cardiovascular events in patients with diabetes mellitus after coronary risk assessment (CRA), prevention of further atherosclerosis or primary prevention in those with a high CRA
Caution eGFR <30 ml/min: reduce dose. Correct hypothyroidism, liver disease, caution in high alcohol intake
Monitoring: NICE 2008: liver function tests (LFTs) before starting and within 3 months and at 12 months of starting therapy. ALT above 3× of normal: stop statin. Do not use if increased risk of rhabdomyolysis and patients should report muscle pain.
Acute porphyria is a contraindication as is pregnancy. Patients should use contraception on statins and one month afterwards to prevent possible teratogenicity. Avoid breast feeding.
SEs: myositis, rhabdomyolysis, pancreatitis is rare, GIT disturbance, hepatitis, jaundice, sleep disturbance, rash and others, very rarely pulmonary fibrosis so patients should report a cough to their GP. If myopathy is suspected and creatine kinase is 5× the upper limit of normal or severe symptoms stop therapy.
How do we know it works? How do we measure this?
Patients’ informed decision-making: Given the evidence, how would the students as doctors present the evidence to a patient? Is the evidence what they would have expected themselves?
Randomised trial of cholesterol lowering in 4,444 patients (35–70 years old) with CHD; patients had angina or previous MI and all on lipid-lowering diet with cholesterol 5.5–8 mmol/L. Double-blind randomised trial to simvastatin or placebo, patient were followed up 5.4 years and simvastatin produced a mean 25% reduction in cholesterol and 35% reduction in LDL (1.3 mmol/L reduction) and 8% increase in HDL.
How many patients died and how many had CHD?
12% (256 pts) placebo group died and 8% (182) in the statin group: 4% improvement in CHD life expectancy in the group over 5 years.
189 deaths from CHD in the placebo group (8.5%) and 111 in statin group (5%) and similar non- CHD deaths, about 50 each group (3.5% improvement in survival)
Placebo risk of surviving over the years was 88% with a 28% chance having another CHD event but this was reduced to a 19% chance in simvastatin group.
After the trial both groups were offered simvastatin and over 80% of each group continued on simvastatin, initially at 20 mg a day. FU 10.4 years total. 414 pts died on statin and 468 who were originally placebo patients died in 10 years. CHD deaths on statin 238 (11%) and 300 on placebo (13.5%). There were 85 cancers on statin and 100 in placebo group with a similar but slightly increased cancer incidence in placebo group.
75% of LDL cholesterol lowering occurs at 20 mg of simvastatin and 6% extra at 40 mg.
The SEARCH Trial showed no increased benefit of 80 mg simvastatin versus 20 mg simvastatin for mortality and morbidity.
Justice to the patient and to society: Students and doctors overestimate the benefits of medications at an individual level but benefits in society are substantial across groups with CHD.
The students would be signposted to relevant resources by the tutor initially.
www.mhra.gov.uk/Safetyinformation/DrugSafetyUpdate/CON199561
Scandinavian simvastatin survival study of 1994 is often called the 4S trial, published in the Lancet
